# Hypocrellin-Mediated PDT: A Systematic Review of Its Efficacy, Applications, and Outcomes

**DOI:** 10.3390/ijms26094038

**Published:** 2025-04-24

**Authors:** Jakub Fiegler-Rudol, Katarzyna Kapłon, Kornela Kotucha, Magdalena Moś, Dariusz Skaba, Aleksandra Kawczyk-Krupka, Rafał Wiench

**Affiliations:** 1 Department of Periodontal Diseases and Oral Mucosa Diseases, Faculty of Medical Sciences in Zabrze, Medical University of Silesia, 40-055 Katowice, Poland; jakub.fieglerrudol@gmail.com (J.F.-R.); rwiench@sum.edu.pl (R.W.); 2 Department of Internal Diseases, Angiology and Physical Medicine, Centre for Laser Diagnostics and Therapy, Medical University of Silesia in Katowice, Batorego 15, 41-902 Bytom, Poland; katarzyna.kaplon.kk@gmail.com (K.K.); kornela.kotucha@gmail.com (K.K.); magdalenamos99@gmail.com (M.M.)

**Keywords:** hypocrellin, photodynamic therapy, antimicrobial resistance, reactive oxygen species, drug-resistant pathogens

## Abstract

Photodynamic therapy (PDT) is a light-activated treatment that generates reactive oxygen species (ROS) to induce microbial cell death. As resistance to traditional antibiotics intensifies globally, PDT has emerged as a promising alternative or adjunctive antimicrobial strategy. Among various photosensitizers, Hypocrellin, a perylenequinone compound, has shown high ROS yield and broad-spectrum activity against bacteria and fungi. This systematic review evaluated the efficacy, safety, and therapeutic potential of Hypocrellin-mediated antimicrobial photodynamic therapy. Following PRISMA 2020 guidelines, a comprehensive literature search was conducted in PubMed, Embase, Scopus, and the Cochrane Library for studies published between 2015 and 2025. Eligible studies included in vitro and preclinical in vivo research using Hypocrellin as a photosensitizer. Quality and risk of bias were assessed using a structured nine-item checklist. Ten eligible studies, all conducted in China, were included. Hypocrellin-mediated aPDT significantly reduced microbial loads in both planktonic and biofilm states of resistant pathogens such as Candida albicans, Candida auris, Cutibacterium acnes, and Staphylococcus aureus. The treatment acted via ROS-mediated apoptosis, membrane disruption, and mitochondrial dysfunction, with minimal cytotoxicity to mammalian cells. Studies also reported enhanced efficacy when Hypocrellin was incorporated into nanocarriers, polymeric scaffolds, or combined with chemodynamic or photothermal therapies. However, substantial heterogeneity was observed in Hypocrellin concentrations, irradiation parameters, and outcome measures. Hypocrellin-based PDT exhibits potent antimicrobial activity and favorable safety in preclinical settings, supporting its potential as an alternative to conventional antibiotics. However, standardized treatment protocols and robust clinical trials are urgently needed to validate long-term safety and translational feasibility. These findings underscore the broader promise of PDT in addressing drug-resistant infections through a mechanism unlikely to induce resistance.

## 1. Introduction

Photodynamic therapy (PDT) is an increasingly recognized, minimally invasive treatment modality that harnesses a photosensitizer (PS), a specific wavelength of light, and molecular oxygen to produce cytotoxic reactive oxygen species (ROS) in target cells [1,2,3,4,5]. Originally developed and extensively studied in oncology and dermatology, PDT has demonstrated efficacy in treating various malignancies, as well as in managing conditions such as psoriasis and macular degeneration [6,7,8]. More recently, its potential has broadened to include a range of clinical applications, including antimicrobial photodynamic therapy (aPDT), where the goal is to eradicate or reduce pathogenic microorganisms [9,10,11,12,13,14,15]. The surge of interest in aPDT has been driven by the relentless rise in antimicrobial resistance, which has emerged as a significant global health concern [9,10,11]. As conventional antibiotics become less effective against drug-resistant pathogens, the ROS-mediated mechanism of aPDT offers a promising alternative for infection control. Unlike systemic antibiotics, the multitargeted oxidative damage inflicted by ROS on microbial cells can limit the development of resistance [9,10].

**Figure 1:**
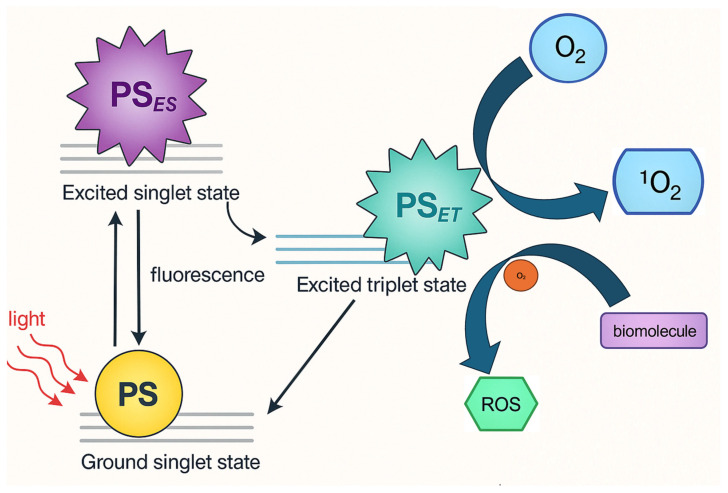
Photophysical Pathways of Hypocrellin as photosensitizer in Photodynamic Therapy: Generation of Reactive Oxygen Species via Type I and Type II Mechanisms.

**Figure 2:**
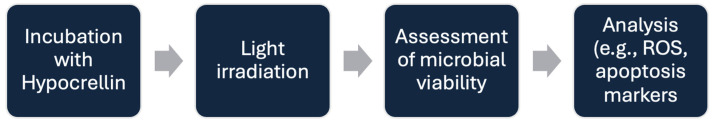
PDT Experimental Setup.

In photodynamic therapy, two primary mechanisms, Type I and Type II reactions, govern the cytotoxic effects induced by activated photosensitizers. In Type I reactions, the excited triplet state of the photosensitizer interacts directly with surrounding biomolecules or molecular oxygen through electron or hydrogen transfer, leading to the formation of highly reactive oxygen species (ROS) such as superoxide anion (O_2_^−^), hydroxyl radicals (•OH), and hydrogen peroxide (H_2_O_2_) [9,10,11,12,13]. These ROS cause oxidative damage to cellular components like membranes, proteins, and DNA. In Type II reactions, the excited photosensitizer transfers energy directly to molecular oxygen (O_2_), converting it into singlet oxygen (^1^O_2_), a highly reactive and short-lived form of oxygen that rapidly oxidizes cellular targets. Both pathways result in cell death, but Type II is generally considered the dominant mechanism in oxygen-rich environments, while Type I may prevail in hypoxic or oxygen-poor conditions [9,10,11,12,13]. These are shown in Figure 1. Figure 2 shows the experimental setup flowchart.

A variety of photosensitizers, such as porphyrins, phthalocyanines, phenothiazines, and perylenequinones, has been investigated for both anticancer and antimicrobial applications [16,17]. Among these, Hypocrellin, a naturally occurring perylenequinone, has gained prominence due to its relatively high singlet oxygen yield, broad-spectrum antimicrobial effects, and comparatively low toxicity toward mammalian cells [12,18,19,20,21,22]. Despite the encouraging results, the broader PDT research community continues to explore pivotal questions surrounding the optimal design and use of Hypocrellin-based PDT. Ongoing debates focus on the most effective drug concentrations, illumination parameters (e.g., wavelength, fluence, and power density), and the merits of combining Hypocrellin with existing antimicrobials or emerging nanotechnology-based delivery systems [23,24,25,26]. Furthermore, there remains a need to clarify long-term safety, any potential off-target effects, and the stability of Hypocrellin-based formulations under diverse clinical conditions. Addressing these knowledge gaps is critical for advancing Hypocrellin-mediated PDT from an experimental approach—whether aimed at tumors or pathogens—to an established, evidence-driven therapeutic option.

### Objectives

The primary objective of this systematic review is to critically evaluate the efficacy, applications, and therapeutic outcomes of Hypocrellin-mediated photodynamic therapy in antimicrobial treatment. By systematically analyzing preclinical and in vitro studies, this review aims to determine the antimicrobial effectiveness of Hypocrellin-based PDT in eradicating bacterial and fungal pathogens, particularly multidrug-resistant strains. This review will also assess variations in treatment parameters, including Hypocrellin concentration, light source wavelength, energy fluence, and irradiation duration, to identify optimal PDT protocols. Additionally, the potential synergistic effects of Hypocrellin-mediated PDT when combined with other antimicrobial agents or nanotechnology-based delivery systems will be examined. A secondary objective is to explore the safety profile of Hypocrellin-mediated PDT by analyzing cytotoxicity data on host tissues, assessing potential off-target effects, and evaluating its applicability for clinical translation. By consolidating current evidence, this systematic review seeks to establish the feasibility of Hypocrellin-mediated PDT as a viable alternative or adjunct to conventional antimicrobial therapies, guiding future research and informing clinical applications in infection management.

## 2. Methods

### 2.1. Focused Question

A systematic review was conducted using the PICO framework [27], defined as follows: In patients with microbial infections (Population), does treatment with Hypocrellin-mediated antimicrobial photodynamic therapy (Intervention), compared to light-based therapy without Hypocrellin, the use of Hypocrellin alone, or conventional antimicrobial treatments (Comparison), result in more effective eradication or reduction in microbial pathogens (Outcome)?

### 2.2. Search Strategy

This systematic review has been registered with PROSPERO under the ID CRD420251027986. The study followed the Preferred Reporting Items for Systematic Reviews and Meta-Analyses (PRISMA 2020) guidelines [28]. A comprehensive literature search was performed across major electronic databases, including PubMed/Medline, Embase, Scopus, and the Cochrane Library. The detailed search strategy is presented in Figure 1. Three independent reviewers conducted database searches using standardized search terms. To refine the selection process, electronic filters were applied to limit results to English-language studies published between 1 January 2014 and 3 December 2024. The initial screening involved reviewing titles and abstracts for alignment with predefined inclusion criteria (Table 1). This was followed by a full-text review conducted by two independent authors to extract relevant data. To broaden the scope of the review, a snowballing technique was used, examining the reference lists of selected studies to identify additional eligible literature. This review aimed to assess the potential of Hypocrellin-mediated antimicrobial photodynamic therapy as an effective strategy for microbial eradication, evaluating its role as a potential alternative or adjunct to standard antimicrobial treatments. The final study selection was guided by clearly established inclusion and exclusion criteria.

### 2.3. Study Selection Process

To uphold methodological rigor and reduce the risk of bias, all retrieved records underwent an independent screening process by multiple reviewers. Titles and abstracts were carefully evaluated against the inclusion criteria, and any differences in judgment were addressed through collaborative discussion until a unanimous decision was reached (Table 2). This structured and transparent approach, consistent with PRISMA 2020 recommendations [28], ensured that only studies of high relevance and methodological integrity were included. By applying this stringent vetting process, we aimed to generate a reliable synthesis of the available evidence regarding the efficacy and clinical utility of Hypocrellin-mediated antimicrobial photodynamic therapy in managing infectious diseases.

### 2.4. Risk of Bias in Individual Studies

To minimize selection bias during the study screening process, titles and abstracts of all retrieved articles were reviewed independently by multiple authors. Inter-reviewer reliability was quantified using Cohen’s kappa statistic to assess the degree of agreement [29]. Any inconsistencies in the inclusion or exclusion of studies were addressed through collaborative discussion, ensuring that final decisions reflected a shared consensus. This structured approach helped safeguard the objectivity and methodological rigor of the review process.

### 2.5. Quality Assessment

The methodological quality of all included studies was independently evaluated by three reviewers, with attention to key aspects of aPDT design, execution, and data integrity relevant to Hypocrellin-mediated interventions. To assess risk of bias objectively, a structured scoring system was applied in which a value of 1 was assigned to each item meeting the criterion (“yes”), and 0 to items not meeting it (“no”). The following nine criteria were used for evaluation:

1. Was the concentration of Hypocrellin used in the aPDT protocol explicitly stated?
2. Was the source or manufacturer of Hypocrellin clearly identified?
3. Was the incubation or pre-irradiation time prior to light activation described in detail?
4. Were full technical specifications of the light source provided (e.g., type, wavelength, power output, fluence, power density)?
5. Was a power meter used to validate the delivered light dose?
6. Did the study include an appropriate negative or untreated control group?
7. Were quantitative outcomes presented using valid statistical analysis?
8. Was outcome reporting complete, with no missing or selectively reported data?
9. Was the study free from apparent conflicts of interest or influence from funding sources?

Each study received a cumulative score out of nine based on the number of “yes” responses. Risk of bias was classified as follows: high (0–3 points), moderate (4–6 points), and low (7–9 points). Final judgments on study quality were made in line with the guidance provided in the Cochrane Handbook for Systematic Reviews of Interventions [30]. Table 3 shows the results of this quality assessment.

### 2.6. Data Extraction

Following consensus on the final list of studies eligible for inclusion, two reviewers independently extracted key data using a standardized approach. Extracted information included bibliographic details (first author, year of publication), study design, type of microbial strains investigated, experimental and control group composition, duration of follow-up (where applicable), primary and secondary outcomes, technical specifications of the light source, concentration and formulation of Hypocrellin used, presence of any adjunctive agents, and the durations of both incubation and light exposure phases.

## 3. Results

### 3.1. Study Selection

**Figure 3:**
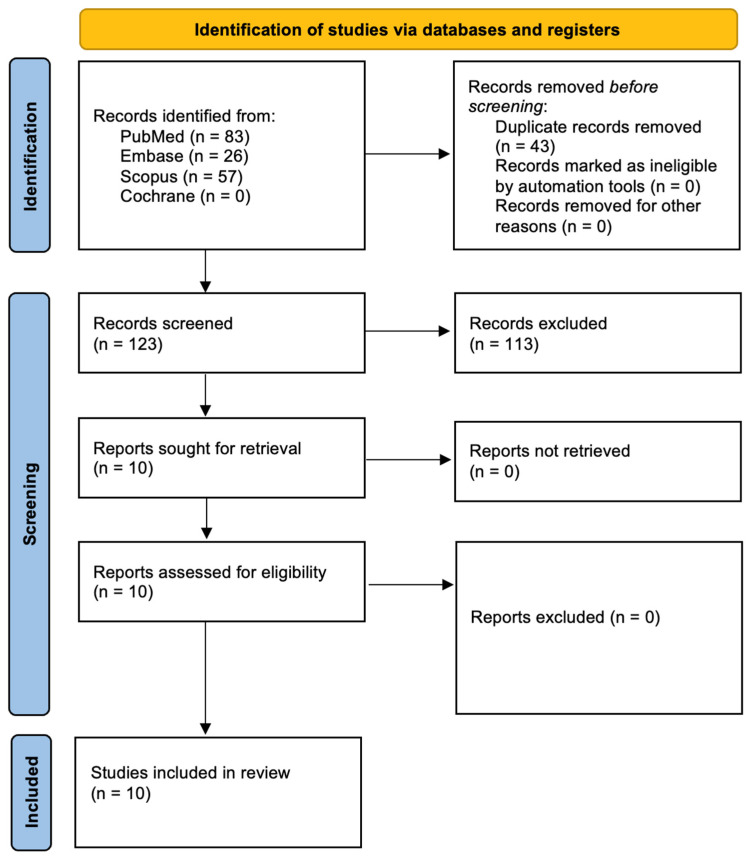
Prisma 2020 flow diagram.

Following the PRISMA 2020 guidelines [28], the study selection procedure is illustrated in Figure 3. An initial database search retrieved 166 records, which were reduced to 10 distinct articles after removing duplicates. Screening titles and abstracts affirmed the relevance of these 10 studies, all of which advanced to full-text review. None were excluded at that stage, leaving a final pool of 10 publications—covering the past decade—to be included in the synthesis. A comprehensive summary of each study’s characteristics and outcomes can be found in Table 4.

### 3.2. Data Presentation

A comprehensive overview of findings drawn from the 10 included studies is presented in Table 4, Table 5, Table 6 and Table 7. These tables supply an organized and reader-friendly synopsis of major results, research procedures, and measured outcomes pertinent to Hypocrellin-mediated aPDT.

### 3.3. Overview of Study Characteristics

Table 4 outlines the core attributes of the studies included in this review, highlighting variations in study design, microbial targets, treatment protocols, and assessment parameters.

### 3.4. Characteristics of Light Sources Used in PDT

Table 6 presents the key physical properties of the light sources utilized in the studies meeting the inclusion criteria. Meanwhile, Table 7 provides a detailed overview of the characteristics of RB across the included studies.

**Table 6:** Characteristics of light sources used.

Author and Year	Light Source	Wavelength (nm)	Energy Density (Fluence) (J/cm^2^)	Power Output (mW/cm^2^)	Irradiation Time (s)
Niu et al., 2020 [31]	LED red light	630	Not stated	5.68	600
Niu et al., 2021 [32]	LED red light	630	3	5.68	Not stated
Yang et al., 2019 [33]	8 W incandescent lamp	400–780	Not stated	Not stated	Not stated
Zhang et al., 2025 [34]	LED light	470	30	Not stated	Not explicitly stated
Jan et al., 2019 [35]	Xenon lamp with optical filter	400–780	72	80	900
Liu et al., 2022a [36]	470 nm laser	470	Not stated	Not stated	1800
Liu et al., 2022b [37]	Laser	470	180	100	1800
Lan et al., 2024 [38]	980 nm NIR laser (via UCNPs upconversion)	Not stated	Not explicitly stated	2000	Variable (up to 1200 s)
Ding et al., 2020 [39]	671 nm laser	671	Not explicitly stated	100	Not stated
Guo et al., 2020 [40]	Laser (assumed, red light)	Not stated	Not explicitly stated	Not stated	Not stated

**Table 7:** Characteristics of Hypocrellin used in studies meeting eligibility criteria.

Author and Year	Hypocrellin Concentration
Niu et al., 2020 [31]	0–1 μM
Niu et al., 2021 [32]	0.25 μM
Yang et al., 2019 [33]	0.5–1.0 μg/mL
Zhang et al., 2025 [34]	1 μg/mL, 0.125 μg/mL, 0.03125 μg/mL
Jan et al., 2019 [35]	0.1, 1, 10, and 100 μM
Liu et al., 2022a [36]	Not stated
Liu et al., 2022b [37]	0.078–2.5 μg/mL
Lan et al., 2024 [38]	0, 5, 10, 50, 100, 200, 300, 400 μM
Ding et al., 2020 [39]	7.7 μM
Guo et al., 2020 [40]	0.69 and 1.38 mg/L, 250 and 500 mg/L

## 4. Discussion

### 4.1. Results in the Context of Other Evidence

Hypocrellin-mediated aPDT demonstrates broad-spectrum efficacy against both bacterial and fungal pathogens, including multidrug-resistant strains such as *Candida auris* and *MRSA*, suggesting its potential as a powerful alternative to conventional antimicrobials [31,36,40]. Upon activation with specific light wavelengths, Hypocrellin generates ROS that induce oxidative damage, apoptosis, and membrane disruption in microbial cells, with minimal toxicity to mammalian cells [33,34]. This mechanism bypasses classical resistance pathways, making it effective against both azole-sensitive and azole-resistant *Candida albicans* [35]. Several studies demonstrated synergistic effects when Hypocrellin is delivered via nanoparticles or polymeric scaffolds, enhancing solubility, cellular uptake, and photodynamic efficiency [38,39]. The addition of chemodynamic therapy (CDT) or photothermal therapy (PTT) further boosts therapeutic outcomes, particularly in hypoxic environments such as tumors or deep infections [38,39]. In vivo models showed reduced microbial loads, accelerated wound healing, and excellent biocompatibility, supporting clinical translational potential [37,40]. Recyclable and light-triggered nanofiber platforms allow repeated applications with sustained efficacy and safety [36]. Despite promising outcomes, optimal light parameters and Hypocrellin formulations vary across studies, highlighting the need for protocol standardization [32,34]. Importantly, Hypocrellin-based aPDT exhibited negligible dark toxicity and strong selectivity for infected tissues, further validating its safety profile [33,35]. Collectively, these findings support Hypocrellin-mediated aPDT as a versatile and effective adjunct or alternative to traditional antimicrobial therapies, especially in the context of rising drug resistance [38,40].

Hypocrellin A has demonstrated significant photodynamic antifungal activity against *Candida albicans*. In vitro studies revealed that HA, when activated by light, induces apoptosis in C. albicans cells through ROS generation, leading to mitochondrial dysfunction and DNA fragmentation. Furthermore, in a murine model of cutaneous *C. albicans* infection, HA-mediated aPDT effectively reduced fungal burden and improved skin lesions without notable toxicity [33]. Similarly, hypocrellin B (HB) has exhibited potent antibacterial effects against methicillin-resistant *Staphylococcus aureus* through sonodynamic action. The mechanism involves ROS production, resulting in significant bacterial inactivation. Notably, HB-mediated sonodynamic therapy did not cause substantial damage to mammalian cells, indicating its potential as a selective antibacterial agent [41]. Advancements in nano-formulations have further enhanced the therapeutic efficacy of hypocrellins. For instance, cage-modified hypocrellin has shown unprecedented activity against multidrug-resistant *Candida* species. This modification improves water solubility and photostability, leading to more effective light-triggered combinational photodynamic therapy [37]. Integrating natural photosensitizers like hypocrellin with nanotechnology has opened new avenues in photodynamic therapy. Nanoconjugated hypocrellin enhances therapeutic precision and efficacy by facilitating targeted delivery and con-trolled activation, thereby minimizing damage to surrounding healthy tissues [42]. The combination of HB and curcumin has demonstrated a synergistic effect in photodynamic inactivation of *Staphylococcus aureus*. This joint action results in a more substantial reduction in bacterial survival rates compared to individual treatments, suggesting a promising approach for controlling foodborne pathogens [43]. In vitro studies have shown that HB effectively inactivates both azole-sensitive and azole-resistant *Candida albicans* strains under light irradiation. This indicates the potential of HB-mediated aPDT as an alternative treatment for resistant fungal infections [35]. Photodynamic therapy has also been explored for treating Staphylococcus aureus infections, with studies indicating its efficacy as an adjunct to traditional antibiotic therapy. This approach could be particularly beneficial in addressing antibiotic-resistant strains [44]. Water-soluble HA nanoparticles have been developed to enhance the photosensitivity and therapeutic potential of HA. These nanoparticles exhibit superior water solubility and photodynamic activity, making them promising candidates for cancer therapy [45]. The production and application of hypocrellins have seen significant advancements, with studies highlighting their excellent light-induced antimicrobial activity against various pathogens, including drug-resistant strains. This positions hypocrellins as valuable agents in antimicrobial PDT [46]. Inactivation of Staphylococcus aureus by HB-mediated photodynamic action has been observed, with notable damage to bacterial cells and increased intracellular ROS levels, leading to cell death. This underscores the potential of HB as an effective photosensitizer in antimicrobial applications [47]. Hypocrellin-based multifunctional phototheranostic agents have been designed for near-infrared (NIR) imaging and therapy of glioblastoma. These agents combine chemotherapy and photodynamic therapy, offering a comprehensive approach to cancer treatment [48]. The development of recyclable, biodegradable, and light-driven antifungal nano-fibrous membranes incorporating HA has shown promise in treating *Candida auris* infections. These membranes provide effective antifungal activity and excellent biocompatibility [36]. Antimicrobial photodynamic therapy (aPDT) has been effective against drug-resistant pathogens, including *Staphylococcus aureus*, *Pseudomonas aeruginosa*, and *Candida auris*. The multi-target mechanisms of aPDT reduce the likelihood of developing resistance, making it a promising alternative to traditional antibiotics [49,50,51,52]. Enhancing the photosensitivity of HA through host–guest complexation with perylene diimide has provided a straightforward approach to improving its photodynamic efficacy. This method offers new avenues for supramolecular theranostics in cancer treatment [53]. The use of polyphenolic natural products as photosensitizers in antimicrobial photodynamic therapy has gained attention due to their safety and compatibility with the human body. These natural compounds offer a promising direction for developing effective antimicrobial treatments [54]. Transferrin-modified cancer cell membrane-coated HB nanocrystals have been developed for targeted cancer therapy. This innovative approach enhances the delivery and efficacy of HB in photodynamic applications [55]. Advancements in the production and application of hypocrellins have highlighted their potential in photodynamic therapy. These natural photosensitizers offer effective antimicrobial and anticancer properties, making them valuable in medical research [46]. The combination of chemotherapy and photodynamic therapy using HA-cisplatin-intercalated hectorite nano-formulations has shown enhanced anticancer efficacy. This synergistic approach offers a promising strategy for cancer treatment [56,57,58]. In vitro studies have demonstrated the effectiveness of HB-mediated photodynamic in-activation against Gram-positive antibiotic-resistant bacteria.

Although Hypocrellin-mediated aPDT has generally demonstrated a favorable safety profile in preclinical models, detailed reporting on specific adverse effects remains limited. Potential systemic responses, such as local inflammation, immune activation, or off-target tissue damage, are rarely quantified or discussed [33,35,36,37,41,42,43,44,45,46,47,48,49,50,51,52,53,54,55,56,57,58]. In particular, the generation of reactive oxygen species (ROS), while central to antimicrobial efficacy, may also elicit oxidative stress in adjacent healthy tissues under suboptimal conditions. Furthermore, long-term immunological consequences, including potential immune modulation or sensitisation, have not been systematically evaluated. Future studies should incorporate comprehensive safety assessments, including histopathological analyses, systemic toxicity markers, and immunological profiling, to fully characterize the risk–benefit ratio of Hypocrellin-based therapies.

### 4.2. Limitations of the Evidence

Despite promising findings, the overall evidence base for Hypocrellin-mediated aPDT remains constrained by several notable limitations. A notable limitation of this review is that all included studies were conducted in China, which may limit the generalizability of the findings to other geographic regions with different microbial profiles, clinical practices, and healthcare infrastructures. Future research should prioritize international collaborations and multicenter studies to validate the efficacy and safety of Hypocrellin-mediated aPDT across diverse populations and clinical settings. Broadening the geographical scope in future reviews would also provide a more comprehensive understanding of regional variations in antimicrobial resistance and treatment response.

First, the included studies exhibit considerable heterogeneity in photosensitizer concentrations, light parameters (e.g., wavelength, fluence, irradiation time), and outcome measures, complicating direct comparisons and consensus on optimal treatment protocols. Second, much of the data derive from in vitro experiments or preclinical animal models, leaving a gap in high-level clinical evidence that would confirm real-world efficacy and safety. Many studies also utilize small sample sizes and short follow-up periods, restricting both the statistical power and the ability to assess long-term outcomes. Additionally, while most reports address basic cytotoxic effects and ROS mechanisms, few systematically investigate potential adverse events or off-target effects, especially in complex physiological environments. Lastly, although several studies highlight the benefits of integrating Hypocrellin with nanocarriers or combining photodynamic therapy with adjunctive modalities, the lack of standardized methods for evaluating synergy and clinical feasibility limits definitive conclusions about their translational potential. Consequently, these shortcomings underscore the need for more rigorous, well-designed clinical trials that incorporate standardized intervention parameters and robust reporting of safety data to fully validate Hypocrellin-mediated aPDT as a reliable antimicrobial strategy.

While the preclinical data on Hypocrellin-mediated aPDT are promising, several key challenges must be addressed to facilitate clinical translation. First, regulatory pathways for light-activated therapies remain complex, requiring rigorous demonstration of safety, efficacy, and device–drug compatibility. Second, formulation standardization is critical; variations in photosensitiser preparation, light dosimetry, and delivery systems across studies hinder reproducibility and clinical scalability. Third, patient acceptability—particularly with regard to photosensitivity, treatment duration, and light delivery methods—requires thorough evaluation in early-phase trials. Addressing these barriers through well-designed translational studies and interdisciplinary collaboration will be essential for advancing Hypocrellin-based aPDT into routine clinical practice.

A further limitation of the included studies is the inconsistent reporting of critical photophysical parameters such as light fluence, power density, and irradiation duration. These variables are essential for reproducibility and comparison across studies. While this reflects a shortcoming in the original publications, it underscores the urgent need for standardized and comprehensive reporting guidelines in photodynamic therapy research. Establishing such standards would enhance transparency, facilitate meta-analyses, and accelerate clinical translation.

### 4.3. Limitations of the Review Process

The primary limitation of our review process arises from the variability among the included studies, which differed considerably in methodologies, intervention protocols, and outcome measures. This heterogeneity made it challenging to conduct a quantitative synthesis; consequently, our findings are based primarily on a narrative summary. We also excluded non-English language publications, introducing a potential language bias that could omit relevant data. Additionally, gray literature and unpublished studies were not thoroughly investigated, further raising the possibility of publication bias. Although we took steps to minimize bias, such as using predefined inclusion and exclusion criteria and conducting independent screenings by multiple reviewers, variations in reporting quality, sample sizes, and study designs may still influence the reliability and generalizability of our conclusions. Finally, short follow-up durations in some studies prevent robust assessment of long-term outcomes, underscoring the need for more standardized protocols and well-designed, large-scale clinical trials to strengthen and refine the evidence base.

### 4.4. Implications for Practice, Policy, and Future Research

The review’s findings suggest that Hypocrellin-mediated aPDT holds considerable promise as an adjunct or alternative to conventional antimicrobial therapies, particularly for drug-resistant infections. For clinical practice, the incorporation of this modality may offer targeted, minimally invasive treatment options; however, standardized protocols regarding light parameters, photosensitizer dosing, and treatment durations are essential before widespread adoption. In terms of policy, healthcare regulators and funding bodies should prioritize and support larger-scale, multicenter clinical trials that rigorously evaluate both efficacy and safety, ensuring that emerging photodynamic therapies are integrated into treatment guidelines based on robust evidence. Future research should also focus on elucidating long-term outcomes, potential synergistic effects with other therapies, and strategies for enhancing delivery systems, all of which could help optimize patient outcomes and contribute to combating the global challenge of antimicrobial resistance.

## 5. Conclusions

The findings of this systematic review indicate that Hypocrellin-mediated antimicrobial photodynamic therapy holds significant promise as both an alternative and an adjunct to conventional antimicrobial treatments. By generating reactive oxygen species upon light activation, hypocrellin demonstrates potent, broad-spectrum antimicrobial activity against various drug-resistant pathogens, while exhibiting minimal toxicity toward host tissues. However, the variability in study designs, photosensitizer dosages, and light parameters underscores the need for standardized protocols and further clinical validation. Future research should focus on optimizing treatment regimens, exploring synergistic combinations with other therapeutic modalities, and confirming long-term safety and efficacy in well-designed, large-scale clinical trials.

## Figures and Tables

**Table 1:** Search syntax used in the study.

Source	Search Term	Filters	Number of Results
PubMed	(hypocrellin) AND (“photodynamic therapy” OR “photodynamic treatment” OR aPDT) AND (antimicrobial OR “microbial infection” OR bacteria OR fungi OR “Candida albicans”)	English language Publication years: 2015–2025 Full text	83
Embase	(‘hypocrellin’:ti,ab,kw) AND (‘photodynamic therapy’:ti,ab,kw OR ‘photodynamic treatment’:ti,ab,kw OR ‘apdt’:ti,ab,kw OR ‘antimicrobial photodynamic therapy’:ti,ab,kw OR ‘photodynamic inactivation’:ti,ab,kw) AND (‘antimicrobial’:ti,ab,kw OR ‘microbial infection’:ti,ab,kw OR ‘bacteria’:ti,ab,kw OR ‘fungi’:ti,ab,kw OR ‘candida albicans’:ti,ab,kw OR ‘c. albicans’:ti,ab,kw)	Publication years: 2015–2025 Controlled Clinical Trial Randomized Controlled Trial	26
Scopus	TITLE-ABS-KEY(hypocrellin) AND (TITLE-ABS-KEY(“photodynamic therapy”) OR TITLE-ABS-KEY(“photodynamic treatment”) OR TITLE-ABS-KEY(“apdt”) OR TITLE-ABS-KEY(“antimicrobial photodynamic therapy”) OR TITLE-ABS-KEY(“photodynamic inactivation”)) AND (TITLE-ABS-KEY(antimicrobial) OR TITLE-ABS-KEY(“microbial infection”) OR TITLE-ABS-KEY(bacteria) OR TITLE-ABS-KEY(fungi) OR TITLE-ABS-KEY(“candida albicans”) OR TITLE-ABS-KEY(“c. albicans”))	Article Publication years: 2015–2025	57
Cochrane	(“hypocrellin” OR “hypocrellin-mediated photodynamic therapy” OR “hypocrellin apdt”) AND (“candida albicans” OR “c. albicans”) AND (“antimicrobial photodynamic therapy” OR “apdt” OR “photodynamic inactivation” OR “photodynamic therapy” OR “photodynamic treatment”)	Publication years: 2015–2025	0

**Table 2:** Selection criteria for papers included in the systematic review.

Inclusion Criteria	Exclusion Criteria
Experimental studies exploring the antimicrobial efficacy of Hypocrellin-based photodynamic therapy, conducted in vitro or using animal models. Research assessing microbial susceptibility to Hypocrellin-mediated aPDT across a range of bacterial or fungal species. Studies in which Hypocrellin is explicitly used as the central photosensitizing agent within the aPDT protocol. Investigations evaluating potential synergistic effects of Hypocrellin-aPDT in combination with established antimicrobial agents. Studies employing controlled designs, including untreated or placebo groups, or comparisons with other antimicrobial modalities. Research directly comparing Hypocrellin-aPDT with conventional antimicrobial treatments in terms of efficacy and/or microbial load reduction. Studies incorporating follow-up periods to evaluate the persistence or recurrence of microbial inhibition following treatment.	Non-academic sources such as conference abstracts, case reports, editorials, opinion pieces, book chapters, and unpublished theses. Studies not subjected to peer review or lacking scientific validation. Publications in languages other than English. Redundant studies, including duplicate reports or articles derived from the same study population without novel data. Research not directly related to infectious disease treatment or that focuses on non-infectious conditions. Studies without a defined comparison or control group to contextualize outcomes. Investigations where aPDT is not applied as an antimicrobial therapeutic intervention. Studies using alternative photosensitizers without direct evaluation of Hypocrellin. Research focusing on unrelated pathogens or general microbial studies lacking specific outcome measures related to pathogen clearance. In vitro experiments conducted under non-representative conditions, limiting clinical relevance.

**Table 3:** The results of the quality assessment and risk of bias across the studies.

Study	1	2	3	4	5	6	7	8	9	Total	Risk
Niu et al., 2020 [31]	1	1	1	1	1	1	1	1	1	9	Low
Niu et al., 2021 [32]	1	1	1	1	0	1	1	1	1	8	Low
Yang et al., 2019 [33]	1	1	1	1	0	1	1	1	1	8	Low
Zhang et al., 2025 [34]	1	1	1	1	1	1	1	1	1	9	Low
Jan et al., 2019 [35]	1	1	1	1	0	1	1	1	1	8	Low
Liu et al., 2022a [36]	0	1	1	1	0	1	1	1	1	7	Low
Liu et al., 2022b [37]	1	1	1	1	1	1	1	1	1	9	Low
Lan et al., 2024 [38]	1	1	1	1	1	1	1	1	1	9	Low
Ding et al., 2020 [39]	1	1	1	1	1	1	1	1	1	9	Low
Guo et al., 2020 [40]	1	1	1	1	1	1	1	1	1	9	Low

**Table 4:** A general overview of the studies.

Author	Country	Study Type
Niu et al., 2020 [31]	China	In vitro
Niu et al., 2021 [32]	China	In vitro
Yang et al., 2019 [33]	China	In vitro
Zhang et al., 2025 [34]	China	In vitro
Jan et al., 2019 [35]	China	In vitro
Liu et al., 2022a [36]	China	In vitro and in vivo
Liu et al., 2022b [37]	China	In vitro and in vivo
Lan et al., 2024 [38]	China	In vitro
Ding et al., 2020 [39]	China	In vitro and in vivo
Guo et al., 2020 [40]	China	In vitro and in vivo

**Table 5:** Main outcomes and details from each study.

Reference Number	Author and Year	Cells Evaluated	Study Groups	Outcomes
[31]	Niu et al., 2020	Human squamous carcinoma A431 cells	Control group—untreated cells. HA alone—cells treated with Hypocrellin A only. Red light alone—cells exposed to 630 nm red LED light only. HA-PDT group—cells treated with HA followed by red light irradiation, representing the experimental photodynamic therapy condition.	HA combined with red LED light inhibited proliferation and triggered apoptosis and autophagy in A431 squamous carcinoma cells. Treatment increased reactive oxygen species (ROS), activating JNK and NF-κB pathways. Inhibiting NF-κB suppressed autophagy and enhanced apoptosis, indicating autophagy’s cytoprotective role. Blocking autophagy further increased apoptosis and NF-κB activity. Antioxidants reversed these effects, confirming the central role of ROS. Study highlights a ROS-mediated, NF-κB-dependent link between apoptosis and autophagy. Supports HA-based photodynamic therapy as a potential treatment for cutaneous squamous cell carcinoma.
[32]	Niu et al., 2021	Primary human keloid fibroblasts	Control group—untreated keloid fibroblasts. HA-only group—treated with HA alone. Red light-only group—exposed to 630 nm red LED light alone. HA-R-PDT group—treated with HA followed by red light irradiation (photodynamic therapy group).	HA-R-PDT significantly reduced proliferation, migration, and ECM accumulation in human keloid fibroblasts (KFs). Induced apoptosis via the mitochondrial pathway. Suppressed autophagy by downregulating TGF-β1/Smad and ERK signaling pathways. Decreased expression of collagen I, collagen III, α-SMA, and fibronectin. Modified MMP/TIMP ratios to favor ECM degradation. Disrupted the bidirectional interaction between TGF-β1 signaling and autophagy, enhancing apoptosis. Suggests HA-R-PDT as a promising antifibrotic therapy targeting the TGF-β/Smad/autophagy/apoptosis axis for keloid management.
[33]	Yang et al., 2019	*Candida albicans*—SC5314 reference strain and two clinical isolates (ATCC18804 and 07318)	Control group—untreated *C. albicans* cells. HA-only group—treated with Hypocrellin A without light. Light-only group—exposed to light without HA. HA + Light group (HA-aPDT)—treated with HA followed by light irradiation. In vivo groups (mouse model): *C. albicans*-infected control (no HA), *C. albicans* infection + 0.5 µg/mL HA + light, *C. albicans* infection + 1.0 µg/mL HA + light.	HA-aPDT effectively inhibited *Candida albicans* growth in vitro and in a mouse skin infection model. Combination of HA and light significantly reduced fungal survival. Increased intracellular ROS disrupted membrane potential and integrity. Induced apoptosis via mitochondrial dysfunction, metacaspase activation, DNA fragmentation, and nuclear condensation. Elevated cytosolic and mitochondrial calcium levels further promoted apoptosis. In vivo, HA-aPDT reduced infection severity and fungal burden without toxicity. Supports HA-aPDT as a safe and effective treatment for cutaneous *C. albicans* infections.
[34]	Zhang et al., 2025	*Cutibacterium acnes*—strain ATCC 11j827 HaCaT human keratinocyte cells	Control group—untreated *C. acnes* cells. HA-only group—*C. acnes* treated with HA without light. Light-only group—*C. acnes* exposed to blue light (470 nm) without HA. HA-PDT group—*C. acnes* treated with HA followed by light irradiation (photodynamic therapy condition).	HA-PDT effectively inhibited *Cutibacterium acnes* in vitro in a light dose- and concentration-dependent manner. Action primarily mediated through ROS-induced type II photodynamic reactions. Disrupted bacterial membrane integrity and induced apoptosis-like features (e.g., DNA fragmentation, chromatin condensation). Caused significant morphological damage to bacterial cells. HA showed minimal cytotoxicity to human keratinocytes at ≤0.5 μg/mL. Indicates HA-PDT as a promising, safe antimicrobial approach for acne treatment.
[35]	Jan et al., 2019	*Candida albicans* ATCC 10231—standard laboratory strain Azole-sensitive clinical isolate Azole-resistant clinical isolate	Control group (P− L−)—no treatment HB-only group (P+ L−)—treated with HB without light Light-only group (P− L+)—exposed to white light without HB HB + Light group (P+ L+)—treated with HB and exposed to light PDT	HB-aPDT inactivated both azole-sensitive and azole-resistant *Candida albicans* strains in vitro. Efficacy was light dose- and concentration-dependent. HB exhibited no dark toxicity. Light activation caused major structural damage: membrane rupture, cytoplasmic condensation, and nuclear disintegration (confirmed via TEM and SEM). Confocal microscopy showed increased HB uptake post-irradiation. Antifungal effect was maintained in drug-resistant strains. Highlights HB-aPDT as a promising alternative for treating resistant *C. albicans* infections.
[36]	Liu et al., 2022a	HeLa cells (a human cervical cancer cell line) for in vitro experiments and 4T1 tumor-bearing mice (a murine breast cancer model) for in vivo studies	In vitro (HeLa cells): Control group—untreated cells NIR-only group—cells exposed to 980 nm near-infrared light without nanoparticles UCNPs@DPB group—cells treated with upconversion nanoparticles loaded with hypocrellin B (HB), without light UCNPs@DPB + NIR group—PDT group: nanoparticles with light UCNPs@DPB-MnO_2_ group—CDT group: nanoparticles with MnO_2_, without light UCNPs@DPB-MnO_2_ + NIR group—PDT + CDT group: full combination treatment In vivo (4T1 tumor-bearingmice): PBS group (Blank control) UCNPs@DPB-MnO_2_ group—CDT treatment only UCNPs@DPB-MnO_2_ + NIR group—combined PDT and CDT treatment under 980 nm laser irradiation	UCNPs coated with HB and MnO_2_ convert NIR light to visible light to activate HB and generate ROS for photodynamic therapy (PDT). MnO_2_ decomposes in acidic, H_2_O_2_-rich environments, releasing oxygen and Mn^2+^ to: Enhance PDT in hypoxic tumors; Enable chemodynamic therapy (CDT) via hydroxyl radical production. In vitro and in vivo studies (HeLa and 4T1 tumor models) showed: Strong antitumor efficacy; High biocompatibility; Tumor-specific accumulation; Minimal side effects. Validates this synergistic PDT/CDT system as a promising treatment for hypoxic cancers.
[37]	Liu et al., 2022b	*Candida auris* (strain BJCA001) fungal cells in both planktonic and biofilm forms. L929 mouse fibroblast cells	In vitro: PLA membrane without light PLA-HA membrane without light PLA membrane with 470 nm light PLA-HA membrane with 470 nm light (PLA-HA-aPDT group) In vivo (rat wound model): *C. auris* infection + PLA membrane *C. auris* infection + PLA membrane + light *C. auris* infection + PLA-HA membrane *C. auris* infection + PLA-HA membrane + light (aPDT treatment group)	Developed a recyclable, light-triggered PLA-HA nanofibrous membrane for aPDT against *Candida auris*. Demonstrated strong antifungal activity in vitro and in vivo. Effectively reduced fungal survival, eradicated biofilms, promoted wound healing, and decreased inflammation. Showed no systemic toxicity and high biocompatibility. aPDT induced intracellular ROS, causing mitochondrial dysfunction, cytochrome C release, metacaspase activation, and nuclear fragmentation—leading to fungal apoptosis. Membrane exhibited strong reusability. Represents a promising alternative for treating superficial fungal infections.
[38]	Lan et al., 2024	*Candida* spp.: *Candida albicans* (HXCB152) *Candida auris* (BJCA001) *Candida tropicalis* (HXCT010) *Candida metapsilosis* (HXCM055) Human keratinocytes (HaCaT cells) and mouse fibroblasts (L929 cells) were also used for cytotoxicity and wound healing evaluations. A rat model was employed to assess in vivo antifungal efficacy and wound healing.	In vitro: Control (no treatment) COP1T alone (with/without light) Free HA alone (with/without light) COP1T-HA (with/without 470 nm light; experimental aPDT group) In vivo (rat wound model): Uninfected control Infected + COP1T (dark) Infected + COP1T (light) Infected + COP1T-HA (dark) Infected + COP1T-HA (light; therapeutic group)	Developed COP1T-HA: a novel cage-modified photosensitizer combining hypocrellin A, a porous organic cage, and polyethylene glycol. Enhanced water solubility and photodynamic efficiency. Showed antifungal activity 6.16× greater than free HA against various multidrug-resistant *Candida* species, including *C. auris*. Effective against both planktonic cells and biofilms. Upon light activation, generated high ROS levels, inducing mitochondrial dysfunction, cytochrome C release, apoptosis, and structural cell damage. In vivo studies in rats showed reduced fungal burden and accelerated wound healing. Demonstrated excellent biocompatibility and minimal toxicity. Highlights COP1T-HA as a promising phototheranostic agent for resistant fungal infections.
[39]	Ding et al., 2020	HeLa cells and 4T1 cells	In vitro study groups (HeLa cells): Control (no treatment) HE or HF only (no irradiation) HE or HF + 671 nm laser (PDT group) HE-PEG-NPs only HE-PEG-NPs + 671 nm laser (PDT at 0.1 W/cm^2^) HE-PEG-NPs + 671 nm laser (PDT + PTT at 0.8 W/cm^2^) HE-PEG-NPs + laser under hypoxic conditions In vivo study groups (4T1 tumor-bearing mice): G1—HE-PEG-NPs + 671 nm laser at 0.8 W/cm^2^ (PDT and PTT) G2—HE-PEG-NPs + 671 nm laser at 0.1 W/cm^2^ (PDT only) G3—HE-PEG-NPs only (no laser) G4—PBS injection (negative control) G5—671 nm laser only (0.8 W/cm^2^, no HE-PEG-NPs)	Developed two novel NIR-absorbing hypocrellin B derivatives (HE, HF) for combined photodynamic (PDT) and photothermal therapy (PTT). Both showed enhanced NIR absorption, strong singlet oxygen generation, and low dark toxicity. HE was formulated into water-dispersible nanoparticles (HE-PEG-NPs). HE-PEG-NPs demonstrated high photothermal conversion efficiency (25.9%) and effective ROS generation. In vitro, exhibited strong tumor cell killing under NIR light, particularly in hypoxic conditions via PDT/PTT synergy. In vivo, enabled tumor-targeted fluorescence imaging and significantly suppressed tumor growth with minimal toxicity. HE-PEG-NPs show strong potential as dual-mode, NIR-activated therapeutic agents for cancer treatment.
[40]	Guo et al., 2020	Methicillin-resistant Staphylococcus aureus	In Vitro Study Groups: MRSA treated with: mPEG-PCL/HA micelles (with and without light) Free HA (with and without light) Blank micelles PBS (control) Vancomycin (positive control) In Vivo (Mouse Model) Study Groups: Mice with induced acute peritonitis by MRSA, treated with: mPEG-PCL/HA micelles at 3 doses: 2.5, 5, and 10 mg/kg HA (with light) Blank micelles (with light) Free HA (5 mg/kg) (with light) mPEG-PCL/HA micelles (5 mg/kg) without light	Developed lipase-sensitive polymeric micelles (mPEG-PCL/HA) for targeted antimicrobial aPDT against MRSA. Encapsulation improved HA’s water solubility and stability. Micelles released HA in response to bacterial lipase at infection sites. In vitro, showed strong antibacterial activity under light, with lower MIC and MBC than in dark conditions. In vivo, achieved 86% survival in a mouse model of acute MRSA peritonitis. Significantly reduced bacterial loads in major organs. Exhibited minimal toxicity and good biocompatibility. Demonstrates potential as a light-activated therapy for superficial drug-resistant bacterial infections.

HA—Hypocrellin A, HB—Hypocrellin B, PDT—Photodynamic Therapy, aPDT—Antimicrobial Photodynamic Therapy, HA-PDT—Hypocrellin A-mediated Photodynamic Therapy, HA-aPDT—Hypocrellin A-mediated Antimicrobial Photodynamic Therapy, HB-aPDT—Hypocrellin B-mediated Antimicrobial Photodynamic Therapy, HA-R-PDT—Hypocrellin A with Red Light-mediated Photodynamic Therapy, ROS—Reactive Oxygen Species, JNK—c-Jun N-terminal Kinase, NF-κB—Nuclear Factor kappa-light-chain-enhancer of activated B cells, ECM—Extracellular Matrix, TGF-β1—Transforming Growth Factor Beta 1, ERK—Extracellular signal-Regulated Kinase, α-SMA—Alpha-Smooth Muscle Actin, MMP—Matrix Metalloproteinase, TIMP—Tissue Inhibitor of Metalloproteinases, TEM—Transmission Electron Microscopy, SEM—Scanning Electron Microscopy, UCNPs—Upconversion Nanoparticles, NIR—Near-Infrared, CDT—Chemodynamic Therapy, MnO_2_—Manganese Dioxide, PBS—Phosphate-Buffered Saline, PLA—Polylactic Acid, COP1T—Cage-Organic Polymer 1T, PEG—Polyethylene Glycol, HE—Hypocrellin B derivative E, HF—Hypocrellin B derivative F, HE-PEG-NPs—HE-loaded PEGylated Nanoparticles, PTT—Photothermal Therapy, MRSA—Methicillin-Resistant Staphylococcus aureus, mPEG-PCL—methoxy Poly (ethylene glycol)-b-Poly (ε-caprolactone).

## Abbreviations

aPDT	Antimicrobial Photodynamic Therapy
ATP	Adenosine Triphosphate
Ca^2+^	Calcium Ion
CDT	Chemodynamic Therapy
COP1T	Cage-Organic Polymer 1T
DNA	Deoxyribonucleic Acid
ECM	Extracellular Matrix
ERK	Extracellular signal-Regulated Kinase
HA	Hypocrellin A
HA-aPDT	Hypocrellin A-mediated Antimicrobial Photodynamic Therapy
HA-PDT	Hypocrellin A-mediated Photodynamic Therapy
HA-R-PDT	Hypocrellin A with Red Light-mediated Photodynamic Therapy
HB	Hypocrellin B
HB-aPDT	Hypocrellin B-mediated Antimicrobial Photodynamic Therapy
HE	Hypocrellin B derivative E
HE-PEG-NPs	HE-loaded PEGylated Nanoparticles
HF	Hypocrellin B derivative F
H_2_O_2_	Hydrogen Peroxide
JNK	c-Jun N-terminal Kinase
LED	Light-Emitting Diode
MBC	Minimum Bactericidal Concentration
MIC	Minimum Inhibitory Concentration
MMP	Matrix Metalloproteinase
MnO_2_	Manganese Dioxide
MRSA	Methicillin-Resistant *Staphylococcus aureus*
NF-κB	Nuclear Factor kappa-light-chain-enhancer of activated B cells
NIR	Near-Infrared
ORCID	Open Researcher and Contributor ID
PBS	Phosphate-Buffered Saline
PDT	Photodynamic Therapy
PEG	Polyethylene Glycol
PICO	Population, Intervention, Comparison, Outcome
PLA	Polylactic Acid
PRISMA	Preferred Reporting Items for Systematic Reviews and Meta-Analyses
PS	Photosensitizer
PTT	Photothermal Therapy
ROS	Reactive Oxygen Species
SEM	Scanning Electron Microscopy
TEM	Transmission Electron Microscopy
TGF-β1	Transforming Growth Factor Beta 1
TIMP	Tissue Inhibitor of Metalloproteinases
UCNPs	Upconversion Nanoparticles
α-SMA	Alpha-Smooth Muscle Actin
μg/mL	Micrograms per Milliliter
μM	Micromolar
mPEG-PCL	methoxy Poly(ethylene glycol)-b-Poly(ε-caprolactone)
